# The Common Swift Louse Fly, *Crataerina pallida*: An Ideal Species for Studying Host-Parasite Interactions

**DOI:** 10.1673/031.010.19301

**Published:** 2010-11-08

**Authors:** Mark D. Walker, Ian D. Rotherham

**Affiliations:** Faculty of Development and Society, Sheffield Hallam University, City Campus, Howard Street, Sheffield, SI IWB, U.K.

**Keywords:** *Apus apus*, avian parasites

## Abstract

Little is known of the life-history of many parasitic species. This hinders a full understanding of host-parasitic interactions. The common swift louse fly, *Crataerina pallida* Latreille (Diptera: Hippoboscidae), an obligate haematophagous parasite of the Common Swift, *Apus apus* Linnaeus 1758, is one such species. No detrimental effect of its parasitism upon the host has been found. This may be because too little is known about *C. pallida* ecology, and therefore detrimental effects are also unknown. This is a review of what is known about the life-history of this parasite, with the aim of promoting understanding of its ecology. New, previously unreported observations about *C. pallida* made from personal observations at a nesting swift colony are described. Unanswered questions are highlighted, which may aid understanding of this host-parasite system. *C. pallida* may prove a suitable model species for the study of other host-parasite relationships.

## Introduction

In order to understand host-parasite systems, the life-history of the parasite species being studied needs to be well known. However, for many parasitic species information about basic biological traits is missing. This lack of knowledge could be hindering a full understanding of host-parasite relationships. Although a number of studies have shown that parasites do have an effect on their hosts (reviewed: Møøller et al. 1990; Lehmann 1990; Møøller 1997) other studies have shown no such effect (e.g. Johnson and Albrecht 1993; Clayton and Tompkins 1995; Lee and Clayton 1995; Eeva et al. 1994). This apparent lack of pathogenicity may be because of a lack of knowledge of parasite life-history.

The common swift louse fly *Crataerina pallida* Latreille (Diptera: Hippoboscidae) may be an excellent example of a parasitic species where no apparent pathogenetic effect has been found, but this may be because of such a lack of detailed knowledge of its life-history. This is an obligate avian nest ectoparasite of the common swift *Apus apus* Linnaeus 1758. However, despite being relatively large, tractable, and having a host species that is common and widely distributed throughout Europe, surprisingly little is known of their biology (Marshall 1981). Much of what is known is scattered among the scientific literature is of substantial age or is in a language other than English, the current hegemonic language of science. Studies have failed to find an effect of its parasitism upon its host (Lee and Clayton 1995; Tompkins et al. 1996).

This is the first review of what is known about this parasite species. This review aims to collate life-history information about *C.* *pallida* and highlight questions requiring further study in order to promote a better understanding of this host-parasite system. New observations made from personal experiences with *C. pallida* from a nesting colony of the common swift situated beneath a roadway bridge close to the town of Olpe, Germany (51°° 04′? 00″? N, 07°° 81′? 00″? E) (Site described by Walker et al. 2009) are described. Several features not previously observed are described.

*C. pallida* may prove to be an excellent model species of a nest ectoparasite, and many of the themes and problems raised may also apply to other host-parasite systems. There are many possible advantages of *C. pallida* as a model nest parasite species, including its large size and easy tractibility, which make conducting experimental work and quantifying levels of parasitism relatively easy compared with other types of nest parasite. It is hoped that this review will prompt investigations of the life-history traits of other host-parasite systems.

### Taxonomy

Louse flies belong to the Hippoboscidae family of cyclorrhaphous insects within the Suborder Brachcera. Hippoboscids are viviparid haematophagous obligate ectoparasites of mammals and birds (Hutson 1984). Formerly the Hippoboscidae were classified along with the bat fly families Nycteribiidae and Streblidae within the single grouping of the Pupipara.

The Hippoboscidae family contains 213 species, and is divided into three subfamilies with 21 genera (Hutson 1984). This family contains a number of well-known and common parasitic species of birds and mammals; for example the avian louse fly *Ornithomya avicularia* from the Ornithomyinae subfamily is a common parasite of a variety of bird species. The Hippoboscinae subfamily contains the horse ked *Hippobosca equine.* The Lipopteninae subfamily contains the deer ked *Lipoptena cervi* and the sheep ked *Melophagus ovinus.* Those species of Hippoboscids that parasitize birds are commonly known as ‘‘louse flies,’’ while those that parasitize mammals, although similar to their avian counterparts, are known as ‘‘keds’’ (Hutson 1984). Most Hippoboscid species occur in the Old World tropics, but 16 species occur in Europe, seven of these on avian hosts (Hutson 1984).

There are eight species within the genus *Crataerina*, three of which occur in Europe. *C. pallida* parasitizes the common swift *A. apus* and the pallid swift *A.* *pallidus*; *C. melba* parasitizes the alpine swift *A. melba;* and *C. hirundinis* parasitizes the house martin *Delichon urbicum.*

### Physical characteristics of *C. pallida*

This species possesses a number of features that aid attachment to its host and reduce the chance of removal through host grooming. It has the standard Arthropod bauplan with there being three tagma —— a distinct head, thorax, and abdomen. The entire body is dorsoventally flattened, which allows it to burrow with ease right to the base of bird feathers and reach its source of food. The exoskeleton is tough, protecting them from being crushed by the host.

The thorax and abdomen are covered with short sharp black hairs, which are also found on the legs and head capsule, and these presumably get caught on the barbs of feathers and provide points of attachment to the host. They are particularly prominent on the posterior abdomen. The joints between the legs are shaped like short sharp hooks, and the legs themselves end in three sharp claws that are ideal for attachment. Adult *C. pallida* have no difficulty in walking upside down across glass or plastic surfaces. The head is sunk into the thorax, and the mouthparts are partially retractable, which protects them from abrasion with the host integument (Lehane 1991).

As for many Hippoboscid flies, *C. pallida* has atrophied vestigial wings that are borne on the thorax and are not capable of sustaining powered flight. A number of Hippoboscid species do retain functional wings, for example the horse ked *H. equine.* Some species lose their wings on finding a host, such as *Allobosca* spp. where the wing tips are lost or the deer fly *L. cervi* where the wings are lost entirely once a suitable host is found (Lehane 1991). *C. pallida* is closely associated with their hosts' nests, and therefore an ability to fly is probably not necessary. The wings have probably not degenerated completely because of their value in providing another type of ‘‘hook’’ to allow attachment to the host.

The head capsule of the Hippoboscidae has become specially adapted for its haemophagous diet, but is nevertheless similar in structure to that seen in the Muscidae (Bequaert 1953). The mouthparts form a distinct prognathous which is found on the ventral midline of the head capsule and ends in a closed sclerotized tube or torma. As in all cyclorrhaphids, there is a cibarial pump. There is a pair of sensory antennae.

*C. pallida* are large insects, with females being larger than males. Fifteen female and 14 male engorged adult louse flies were measured during July 2008. The 15 engorged females had a body length of 7.43 mm (SD ±± 0.455), average abdomen width of 5.45 mm (SD ±± 0.53), and abdomen length of 4.01 mm (SD ±± 0.36). Males were smaller with an average body length of 7.16 mm (SD ±± 0.49), abdomen width of 3.78 mm (SD ±± 0.41), and abdomen length of 4.58 mm (SD ±± 0.42). This difference in size is not simply due to the fact that females can store a larger volume of blood. Females have been found to be larger than males both in the engorged and unengorged states (Kemper 1951). Females probably have to be larger than males as they are the sex which produces eggs and provision the larvae internally.

The legs are held away from the body when at rest, and this gives *C. pallida* a characteristic ‘‘spider’’ or ‘‘star-like’’ stance. In colouration, the adult imagines are a light to dark brown colour. Teneral specimens have a translucent sheen, which is, however, soon lost. In imagines that have fed, the abdomen is noticeably larger and more swollen and is a light to dark grey colour. *C. pallida* with dark red coloured abdomens are occasionally seen, and these have presumably recently fed.

Differentiating between the sexes of engorged *C. pallida* is easily done with the naked eye (Kemper 1951). In males, a black, semi-circular ring is present on the rear of the abdomen. Females instead have two spot-like triangular black marks. Females have much larger, wider, more engorged abdomens than the males. Males are hairier than females. Discriminating between males and females that have not fed is more difficult. Males have more heavily segmented abdomens than the females, but a magnifying scope is needed to see this. The genitalia of male *C. pallida* can be exposed by gently pressing on the abdomens of the males and thus facilitating sexing.

**Figure 1.**
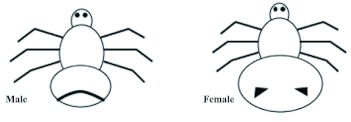
Schematic diagram showing differences between *Crataerina pallida* sexes after Kemper (1951). High quality figures are available online.

### Lifecycle

**Figure 2.**
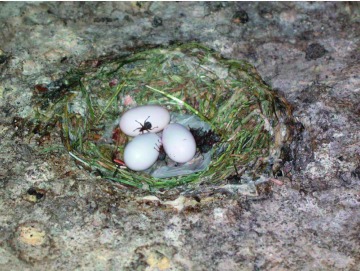
Adult *Crataerina pallida* at the nest during the incubation period of the *Apus apus* eggs. High quality figures are available online.

**Figure 3.**
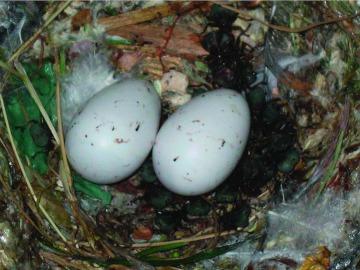
A nest particularly heavily parasitized by adult *Crataerina pallida.* There are approximately 20 adult *C.* *pallida* in this nest. High quality figures are available online.

There is a strong association between the lifecycle of *C. pallida* and that of the host's breeding season. 4^th^ instar imagines emerge synchronously with the return of the common swift in spring. Pupae are cyclorrhaphous. Although emergence has been found to coincide with the hatching of swift nestlings (Büütiker 1944; Lack 1956), others have found that it occurred earlier (Hutson 1981; Bromhall 1980). In 2007, the first *C. pallida* emerged on 15 May, during the period of swift egg laying (e.g. Figures 2 and 3). In 2008, *C. pallida* had emerged before the 03 June, when nestlings began to hatch. Weather conditions may influence the exact timing of emergence of *C. pallida.*

The emergence from the pupae appears to be temperature mediated. Anecdotal reports suggest that pupae left on a radiator began to hatch after several days (Kemper 1951). In a more analytical study, emergence of the house martin louse flies occurred more rapidly at elevated temperatures (Popov 1965).

Sixty pupae were collected from a *C. pallida* colony in 2008 and divided into three groups of 20 pupae, and from 03 October, each group was kept either continuously at room temperature at approximately 20°°C, within a refrigerator (mean temperature 5°°C), and within one of two warm cabinets (mean temperatures of 24 and 47°°C).

Adults emerged from 9 of the 20 pupae kept at room temperature between 27 April and 02 May, somewhat earlier than what would be expected. The group kept in the refrigerator hatched between August and October the following year, much later than would occur under normal conditions. Emergence occurred at none of the pupae kept in the warmest warm cabinet, probably because the temperatures experienced were lethal. However, emergence of those kept in the cooler warm cabinet occurred between the 27 and 30 April with *C. pallida* emerging from 7 of 20 pupae. Six of 20 pupae kept in a refrigerator for 3 months from August to November in order to simulate ‘‘winter,’’ and thereafter at room temperature to simulate ‘‘spring’’, began to hatch in mid-February, roughly three months earlier than normal, thus indicating that a period of winter cooling may be necessary for emergence to occur.

Mating of *C. pallida* usually takes place on or in close proximity to the nest, but may also occur on the adult or nestling swifts. As in bat flies (Strebilidae and Nycteribiidae), blood ingestation may be necessary for succesful copulation to occur (Yuval 2006). Mate guarding seems to occur, with male *C. pallida* sometimes remaining mounted on the females for several minutes at a time. Two or three *C. pallida* males may attempt to mount a single female. Mating competition may increase as the summer progresses perhaps due to the limited amount of time available before swift departure and due to the falling number of females. ‘‘Clusters’’ of *C. pallida* often occur in which more than 20 *C. pallida* may congregate together in one large mass (Figure 3).

Female *M. ovinus* are able to store enough sperm after a single mating to fertilise all their subsequent eggs (Evans 1950; Small 2005). Should this prove to be the case with *Crataerina* species, it might mean that males able fertilise females first could be at a significant advantage than later emerging males. This may explain why males hatch from the winter diapause earlier than the females. It may also help explain the female dominated sex ratios seen during the summer, as there may be no advantage for males in staying alive after they have copulated. Their presence may increase the parasitic burden on the hosts that their own offspring will ultimately rely on.

**Figure 4.**
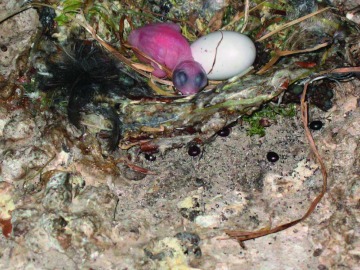
Pupae deposited to the side and beneath the nest. Two of the pupae are a dark brown in colouration, indicating that they have only recently been deposited. Pupae are typically black in colouration. Beneath the nest to the right is a small aggregation of adult *Crataerina pallida* that may be the result of mating competition. High quality figures are available online.

Larvae develop singly within the female's uterus in a mechanism known as adenotrophic viviparity. Larvae are nourished through special milk glands found within the common oviduct (Baker 1967) and, if development is similar to that of other Hippoboscid species, takes approximately 3 weeks (Small 2005). Larvae are deposited when they reach the third instar, and they then pupate almost immediately (Baker 1967). Larvae are deposited either underneath or some distance away from the nest (Figure 4). In comparison, other Hippoboscidas deposit pupae at no specific location, for example those of the genus *Lipoptera*, or the pupae are purposely attached to the host as is the case in *M. ovinus* (Lehane 1991). On deposition, pupae are a light brown colour and require six hours to become hardened and dark in colouration.

Hippoboscids have relatively low fecundity. It is unknown how many larvae a single female can produce, but female sheep keds can produce new larva every 6 to 8 days, and so can therefore probably produce between 12 and 15 larvae over the course of a lifetime (Small 2005). A similar figure in Crataerinids is likely. Other Hippoboscids have lifespans of between 6 and 10 weeks (Lehane 1991; Small 2005). The number of pupae seen at the nest has been found to be higher at the end of July than in June (Kemper 1951). This indicates that most pupal production occurs during the month of July, during the nestling period. Pupae remain in diapause until the following spring.

Basic life-history information about *C. pallida* is missing, for example information on the lifespan of adults, the number of pupae females are capable of producing, and the factors affecting adult emergence each spring.

### Population dynamics

**Population size.** At the study site, the population of *C. pallida* found at the nests during 2007 peaked during mid-May, which coincided with the incubation of the eggs. In 2008, *C. pallida* numbers peaked during the incubation and were falling by the time the nests could be first examined at the end of incubation. Throughout the nestling period of both years, the number of *C. pallida* seen steadily dropped. A similar pattern has been reported for *C. hirundinis* (Bequaert 1953). Studies on the number of *C. pallida* on captured adult birds also show a decrease in numbers as the summer progresses (Hutson 1981).

*A. apus* pairs are nest-site faithful, often returning year after year to the same nest site (Weitnauer 1947; Lack 1956). This may affect *C. pallida* populations, allowing them to increase on a year by year basis at individual nests with progressive use. At the study site, new and young nests do appear to be less heavily parasitized than more obviously older, well-established nests, although not enough time has passed to show this conclusively. It may be the case that a build-up of parasite numbers over several years may be a factor causing nest abandonment and the establishment of new nests in an attempt to forego parasitism.

Other factors, such as the weather or climate, may also influence *C. pallida* numbers. A correlation between the abundances of a louse fly species on Serins, *Serinus serinus*, and the weather has been seen (Summers 1975). Recently fledged nestlings of the north island robin *Petroica australis* were more likely to be parasitized by *C. pallida* if they came from wetter territories (Berggren 2005).

The number of *C. pallida* seen at particular nests can vary considerably on a day by day basis. This may be due to *C. pallida* moving onto and off the adult hosts and thus being removed temporarily from the nests. This, along with the general changes in *C. pallida* numbers that occur throughout the swift breeding season may lead to a false picture of the true intensity of parasitism being made if the population is sampled on only a small number of occasions. Data on the consistency of *C. pallida* populations over the entire season and on a day-by-day basis is needed.

Another factor which may influence the population size of *C. pallida* seen at a nesting colony is the size of the colony involved. Generally speaking larger nesting aggregations of birds are more heavily parasitized. Whether this occurs with *Crataerina* spp. is difficult to decipher, as relatively few colonies have been studied. The population of *C. pallida* seen at the well studied Oxford colony of the common swift is smaller in size than that seen at the study site despite the fact that it houses considerably more nesting swifts.

Host predation may be a major cause of Hippoboscid mortality (Hutson 1984). However, this is not the case for *C. pallida.* Adult *A. apus* are reported to ignore adult louse flies and to take no measures to remove them from themselves (Lack 1956; Bromhall 1980). *A. apus* nestlings do not feed on adult *C. pallida.* Should an *A. apus* manage to preen a *C. pallida* with its beak, the parasite will simply wait until the bird opens its mouth and crawl out (G. Candelin personal observation). Ironically, *C. pallida* may be the prey of a parasitic wasp. Two species of Hymenoptera of the Pteromalidae family, *Nasonia vitripennis* and *Dibrachys cavus* have been reared from the puparia of *C. pallida* and maybe also *C. hirundinis* (Bequaert 1953).

**Aggregation and prevalence.** Parasitic species typically exhibit aggregated population distributions. This is the case for *C. pallida* (Hutson 1981) and for *C. melba* (Tella & Jovani 2000), although the level of aggregation seen by these species is lower than seen in other host-parasite systems.

The prevalence of parasitism exhibited by louse fly species is much higher than is normally seen in other parasites. On adult alpine swifts infestation rates by *C. melba* of 70.8% (Tella and Jovani 2000) and of 74% (Tella et al. 1995) averaged over the summer were found. On *A. apus* adults parasitized by *C. pallida* the average infestation over the entire season was 34.4% (Hutson 1981), and at *A. apus* nests 67% (Tompkins et al. 1996). For comparison, the prevalence of the louse fly *Ornithomyia avicularia*, on serins *S. serinus*, was found to be 3% (Senar et al. 1994), and the prevalence of other Hippoboscid flies on other species has been shown to be no greater than 20% (McClure 1984).

The infestation rate of adult swifts has been found to vary with date, being at around 10% in early spring, raising quickly to 50% during the incubation period, and reaching a maximum of 50% to 60% around the time of nestling hatching, before declining rapidly during the second period of nestling growth (Hutson 1981). These changes can probably be explained through changes to the *A. apus* lifecycle, with infestation being highest during incubation when *A. apus* are at the nest for the longest periods, and falling when they are feeding the young and are there less often. It has been proposed that the high prevalence of louse flies on swifts could be due to their short legs and lack of easily moveable head, which prevents birds from effectively removing parasites (Tella et al. 1998).

The prevalence of *C. pallida* and their intensity of parasitism has been determined at only one nest site, at the Oxford University Museum site used in the original study by Lack (1956). At this study site, a mean parasitic intensity of only one adult *C. pallida* per nest has been found, with the maximum number in any one nest being 9 adult *C. pallida* (Lee and Clayton 1995). At the study colony, where nests are left in place between breeding seasons, the maximum number of *C.* *pallida* seen in a single nest in 2007 was 27, and the average number of *C. pallida* seen per nest was 3.64 (SD ±± 2.65). These figures are substantially higher than those seen at Oxford. However, it is usual at the nesting site at the museum for nests to be removed on a yearly basis (G. Candelin personal communication). This may lead to a distortion of louse fly populations and to an artificially lower number of parasites per nest than would normally occur. It has been shown that the removal of old, heavily parasitized nests affects the distribution and intensity of parasitism in nest box studies (Møøller 1989). The removal of nests and the resulting unnaturally lower levels of parasite abundance seen may be the reason why studies at Oxford failed to find any negative costs of *C. pallida* parasitism.

**Sex ratio.** Louse fly populations are female-biased. More female than male *C. hirundinus* were found at house martin nests and on adults (Summers 1975; Popov 1965; Hardenberg 1929); likewise for *C. melba* at alpine swift nests (Tella and Jovani 2000). A greater proportion of female than male *C. pallida* has been seen on adult *A. apus* (Hutson 1981). This female bias is puzzling as an equal number of males and females are thought to hatch (Bequaert 1953). Other Hippoboscids, such as *M. ovinus*, have more equal sex ratios (Small 2005). Distinct differences in the sex ratio at different stages of the summer have been found (Kemper 1951). In spring, female *C. pallida* were seldom found on adult *A. apus.* The proportion of males found dropped rapidly as incubation began. This may be due to males emerging and then dying off before females (Kemper 1951). This idea tallies with observations of pupae in the lab, where males consistently emerged first.

Tella and Jovani (2000) found that the ratio of male and female *C. melba* louse flies on hosts was inter-connected with mate attraction being one possible cause. As mating competition appears to be strong in *C. pallida*, this may also be a factor influencing sex ratios and population dynamics. The effect of such mate attraction as a factor affecting parasite population biology, and thus pathogenicity, has rarely been looked at, and this species may therefore prove an ideal model species for such studies.

**Transmission and dispersal.** When adult *A. apus* return from overwintering sites in Africa, they are *C. pallida*-free (Zumpt 1966). Therefore, an easy way for *A. apus* to avoid *C. pallida* parasitism would be to build a new nest in a *C. pallida*-free place. Where *C.* *pallida* have been marked, it has been seen that although *C. pallida* could move between nests, this rarely occurred, with only 6 from 96 flies moving to adjacent nests (Summers 1975). Whether this was active dispersal by *C.* *pallida* themselves or whether they were carried between nests could not be determined. *C. pallida* have no mechanism themselves to move between nests discretely separated from each other or to new colonies some distance away from existing ones. Transmission has been assumed to be vertical based on these results (Lee and Clayton 1995; Tompkins et al. 1996). However, this study showed only that *C. pallida* are unlikely to move to other nests under their own locomotion and did not preclude them being carried to other nests by nestlings or adult *A. apus.*

During the breeding season when the nestlings are at the nest, transmission is undoubtedly vertical. However, once the nestlings fledge, they can be no longer be re-infected with *C. pallida* from the natal nest, and when they return from the winter migration, they are *C. pallida*-free. Thereafter, transmission of *C.* *pallida* must be horizontal and occur from adult to adult, or from adult to nest to adult. Most likely is that *C. pallida* are transmitted to new sites through adult *A. apus* or first year adults that visit new or existing nest sites and carry *C. pallida* with them. A greater proportion of female than male *C. pallida* were found on adult house martins (Summers 1975), which may be the result of females feeding more often than males, but could also be because gravid females actively transfer onto adults as doing so they may be dispersed to new sites where they can deposit their pupae. Females acting in such a way as to facilitate their own dispersal would increase their lifetime reproductive success if they managed to get transferred to a new formerly uncolonised nest site which they and their offspring could successfully inhabit without experiencing intra-specific competition.

### Parasitism

**Pathogenicity.** No pathogenic effect of *C. pallida* parasitism on their *A. apus* hosts has been found (Lee and Clayton 1995; Tompkins et al. 1996; Hutson 1981). This is surprising. *C. pallida* feed once every 5 days, males taking 23 mg, and females 38 mg of blood (Kemper 1951). It has been calculated that if the total blood volume is estimated as being 10% of total body weight; then in an adult *A. apus* weighing 42 grams, this represents about 5% of its blood being lost (Campbell 1988). Therefore, substantial quantities of blood may be lost.

Adult *A. apus* with heavy infestations had weights within the normal weight range of adult swifts leading to one author to conclude that there was no evidence that heavy *C. pallida* infestation affected adult condition (Hutson 1981). There are anecdotal reports of grounded *A. apus* having *C. pallida* (Büütiker 1944; Lack 1956); however, this is hardly strong evidence for a negative effect of these parasites. No correlation between *C. pallida* intensity and nestling body mass, the fledgling date, or the number of chicks fledged from each nest has been found (Lee and Clayton 1995). Where *C.* *pallida* abundances were artificially manipulated, no differences in nestling growth or fledging success was seen (Tompkins et al. 1996). Although no pathogenic effect has been found on *A. apus*, a number of studies have found an adverse effect of the closely related louse fly, *C.* *melba*, on the Alpine Swift (Bize et al. 2003; Bize et al. 2004; Bize 2005).

The type and level of transmission and transfer of parasites between hosts is important in influencing the level of parasite virulence seen (Bull 1994). Parasites that transfer between hosts in a mainly vertical manner, from parent to offspring, typically exhibit lower levels of pathogenicity than parasites that transfer between unrelated hosts horizontally (Eward 1994). Tompkins et al. (1996) postulated that the lack of virulence seen by *C. pallida* may be due to the vertical nature of its transmission. However, horizontal transmission also occurs and is commonly reported at colonies where nests are situated close together (e.g. Bize et al. 2003; Bize et al. 2005). *C. pallida* which have not fed have been shown to be more active than those that have (Miller 1997), and thus may be more likely to transfer between closely situated nests where these are available. The pathogenicity of *C. pallida* may be dependant and may alter depending on the nature of the nest colony at which it is found; because of this *C. pallida* may prove an interesting model species for looking at the evolution and development of parasite transmission and pathogenicity.

By looking for more subtle effects of parasitism, such as compensatory growth during the nestling phase, the sex ratio of fledging nestlings, and the lifespan and reproductive success of adult parent birds effects of parasitism by *C. melba* on the Alpine Swift have been found (Bize et al. 2003; Bize et al. 2004; Bize et al. 2005). Saino et al. (1998) found that the speed of growth of Barn Swallow nestling wings was influenced through parasitism by the *O. biloba* louse fly. Future studies investigating *C.* *pallida* parasitism should likewise look at such finer aspects of *A. apus* reproductive success and not simply on the more obvious parameters such as adult weight, nestling fledging weight, and nestling survival, as has been before. More direct effects of parasitism, such as parasite caused anaemia, have yet to be reported but are likely to occur as a result of the blood loss experienced by hosts parasitized by *C. pallida.*

**Mode of parasitism.** Crataerinid louse flies, unlike other types of louse flies such as *O. avicularia*, are monoexous, being host specific (Kemper 1951; Tella and Jovani 2000). However, in addition to parasitizing *A. apus*, *C. pallida* is also reported to parasitize the pallid swift (M. Cucco personal communication). The development of host specificity within louse fly-avian parasite systems may be worth investigating further. Is there any separation in the Crataerina populations parasitizing Common and Pallid Swifts? Could divergence occur in the future?

When initiating feeding, *C. pallida* dive between the feathers to reach the skin. Feeding *C. pallida* appear somewhat like ticks, with the heads being burrowed into the host, while the legs and abdomen protrude outwards. When they finish feeding, they move backwards away from the skin of the host, before delving into a new position to feed. On nestlings, they are often found feeding on the lower rump area. On adults, they are reported to feed preferentially on the belly and neck (Kemper 1951). *C. pallida* which have not fed have abdomens that are noticeably smaller and have a light brown colouration. In adults that have fed, the abdomen is substantially larger and has a greyish colouration.

**Host selection.** When faced with a brood of chicks parasites have to choose one, and they may be different. Although large nestlings may offer large resources, they will have strong immune responses; weak nestlings on the other hand will offer fewer resources but will be less able to invest in immune defences (reviewed: Sheldon and Verhulst 1996). Louse flies are an ideal parasite to study these trade-offs. Host preference of *C. melba* has been found to be linked to nestling age, preferring older siblings with more developed feathers (Roulin 2003). Later when there was little difference in feather development between nestlings, these preferences disappeared and no nestling was favoured. Conversely, a later study found that nestlings intermediate in size were preferred, perhaps a compromise choice between nestling resources and immune response (Bize et al. 2008).

Typically host-parasite studies consider the effects of parasitism on the level of the individual. However, in the case of *C. pallida*, and maybe other nest parasites, a more appropriate level of study may be to consider each nest, with its associated parent and nestling birds, as being a discrete unit of parasitism. Attempts should also be made to try to explain features of parasite life-history in relation to their hosts and their hosts' life-histories. A parasite's life-history features may be tuned to those of its host, thus enhancing parasite fitness. To what extent are the skewed sex ratios, the declining population sizes, and the intense mating competition exhibited by *C. pallida* the result of *C. pallida* attempting to maximise their fitness in the face of the biology and breeding biology of their avian hosts? Future studies should consider aspects of parasite life-history as being adaptations to the host species on which they prey.

**Vectors.** It is known that Hippobiscid flies act as vectors of various species of Trypanosoma and Haemoproteus (Baker 1967; Bize et al. 2005). *Crataerina* spp. may also act as vectors of such parasites and such a role has been discussed (Soulsby 1968). *C. pallida* may engage in a phoretic association with feather mites (Astigmata), and thus aid their transmission (Jovani et al. 2001). Small numbers of feather mites have been found on louse flies collected from avian hosts (Hill et al. 1967). However, studies testing whether this could be the case have found no evidence that such ‘‘hitch hiking’’ occurs (Philips and Fain 1991).

## Summary

The common swift louse fly, *C. pallida*, is a fascinating example of an avian nest parasite, with many puzzling life-history features. When trying to understand parasite life-cycles and ecology it is important to consider what is occurring to the host species and how this may be affecting the parasite, or in what way the parasite may be using the hosts own ecology to its own advantage. Considering the *C. pallida* from this perspective may lead to a better understanding of the strategies it uses.

The common swift louse fly *C. pallida* may prove to be an excellent model species for studying host-parasite systems. It offers a number of advantages to the parasite researcher including large size and the ease at which it can be manipulated. In comparison with other nest and avian parasites, its populations can be easily quantified and determined. *C. pallida* may also prove an excellent example of how hosts and parasites co-adapt, with the life cycle of *C. pallida* appearing to be well in tune with that of their hosts. Connecting parasite life-cycles to that of their hosts may lead to a better understanding of a wide range of host-parasite systems.
